# Predicting Fall Counts Using Wearable Sensors: A Novel Digital Biomarker for Parkinson’s Disease

**DOI:** 10.3390/s22010054

**Published:** 2021-12-22

**Authors:** Barry R. Greene, Isabella Premoli, Killian McManus, Denise McGrath, Brian Caulfield

**Affiliations:** 1 Kinesis Health Technologies Ltd., D04 V2N9 Dublin, Ireland; killian.mcmanus@kinesis.ie; 2 Biomarker Department, Division of Experimental Medicine, H. Lundbeck A/S, 2500 Copenhagen, Denmark; ISPR@lundbeck.com; 3 Department of Basic and Clinical Neuroscience, Institute of Psychiatry, Psychology and Neuroscience, King′s College London, London SE5 9RX, UK; 4 Insight Centre for Data Analytics, University College Dublin, Dublin, Ireland; b.caulfield@ucd.ie; 5 School of Public Health, Physiotherapy and Sports Science, University College Dublin, Dublin, Ireland; denise.mcgrath@ucd.ie

**Keywords:** inertial sensors, Parkinson’s disease, falls, digital biomarkers, gait, Timed Up and Go

## Abstract

People with Parkinson’s disease (PD) experience significant impairments to gait and balance; as a result, the rate of falls in people with Parkinson’s disease is much greater than that of the general population. Falls can have a catastrophic impact on quality of life, often resulting in serious injury and even death. The number (or rate) of falls is often used as a primary outcome in clinical trials on PD. However, falls data can be unreliable, expensive and time-consuming to collect. We sought to validate and test a novel digital biomarker for PD that uses wearable sensor data obtained during the Timed Up and Go (TUG) test to predict the number of falls that will be experienced by a person with PD. Three datasets, containing a total of 1057 (671 female) participants, including 71 previously diagnosed with PD, were included in the analysis. Two statistical approaches were considered in predicting falls counts: the first based on a previously reported falls risk assessment algorithm, and the second based on elastic net and ensemble regression models. A predictive model for falls counts in PD showed a mean R^2^ value of 0.43, mean error of 0.42 and a mean correlation of 30% when the results were averaged across two independent sets of PD data. The results also suggest a strong association between falls counts and a previously reported inertial sensor-based falls risk estimate. In addition, significant associations were observed between falls counts and a number of individual gait and mobility parameters. Our preliminary research suggests that the falls counts predicted from the inertial sensor data obtained during a simple walking task have the potential to be developed as a novel digital biomarker for PD, and this deserves further validation in the targeted clinical population.

## 1. Introduction

Parkinson’s disease (PD), a progressive neurodegenerative disease, has significant deleterious effects on gait and balance. The prevalence of PD has been estimated as 0.3% in industrialized countries [1], increasing with age to 1% in the over-60s, rising further in the over-80s. The costs associated with Parkinson’s disease are significant, estimated to be $23 Bn per year [2,3] in the US and £449 M-£3.3 Bn per year in the UK [4].

The clinical manifestation of PD is characterized by a broad spectrum of motor and non-motor symptoms [5], and the well-recognized four cardinal features of PD are tremor at rest, rigidity, akinesia (or bradykinesia) and postural instability. A clear symptom of the disease, known as Parkinsonian gait, is characterized by slow, shuffling steps, coupled with impaired dynamic balance.

People with PD are at a much higher risk of falls compared to the general population [6]. They are also twice as likely to fall as patients with other neurological conditions [7,8], falling more frequently particularly in the advanced stages of the disease. It has been estimated that 38–68% of PD patients will fall at some point during the course of their disease [6,9,10,11]; however, a range of novel medications and non-pharmacological interventions are under development to address this unmet need [12,13,14,15]. The current clinical evidence suggests the best predictor of a fall in a person with PD is the occurrence of a fall in the previous year [16]. As a result, some clinical trials are adopting self-reported falls during a follow-up period as the primary endpoint [12]. When falls are used as an outcome measure, longitudinal studies with longer durations are needed to obtain sufficient falls data to detect a drug compound’s effect when compared to the baseline. Furthermore, a lengthy study burdens the patients and may delay the time to market for a compound or intervention that could potentially reduce the frequency of falls.

Accurately measuring the number of falls per patient in a clinical trial can be challenging. Data collection usually involves self-reporting either via diaries or regular (e.g., weekly) investigator follow-up. Self-reporting is prone to bias as it relies on patient recall and can be affected by the individual’s perception of a fall [17]. In addition, inaccurate reporting of falls is well-documented [18]. Fall detection technologies use body-worn or ambient sensors to measure or detect the impacts associated with falls. While these systems have been shown to be accurate and sensitive in detecting falls in situations where young adults simulate falls under controlled conditions [19,20], in real-world settings, they have been shown to suffer from a significant rate of false positives. Moreover, they are prone to noncompliance with regard to the long-term wearing of the device [19,21].

The assessment of falls risk in PD patients by measuring individual predictors of falls, such as pathological gait or impaired balance, provides an opportunity to obtain an indication of each patient’s individualized risk of falling [22,23,24,25,26]. In contrast to directly detected falls, falls risk can be assessed frequently, objectively and reliably using clinical tests quantified using wearable sensors in a clinical setting or potentially under free-living conditions [24,27,28,29,30,31]. Moreover, utilizing falls risk assessment as an outcome measure has the potential to reduce the trial sample size and duration. The rate or number of falls observed in PD patients during a clinical trial is often used as a primary outcome measure to capture meaningful, interpretable change attributable to an intervention. However, a digital biomarker predicting the number of falls is currently lacking.

A more promising approach is to use wearable sensors to provide a more sensitive and objective assessment of the response to intervention than what is currently offered by typical functional tests (such as the TUG (Timed Up and Go) test, 180° turn test, the Tinetti Scale [32], the Functional Reach Test and the Berg Balance Test [33]). As an example, the TUG is a standard clinical test of mobility where patients are observed and timed as they rise from a chair, walk 3 meters, turn, walk back to the chair and sit back down. The time taken to complete the test (TUG time), measured using a stopwatch, is compared with standard values with longer times associated with a greater risk of falls. However, studies using TUG time to distinguish fallers and non-fallers in PD patients report only moderate sensitivity [22,34,35], with similar results reported for other standard functional tests [36,37].

Previous research from our group has shown promising results when instrumenting the TUG test with inertial sensors (QTUG), combining signal processing and machine learning algorithms to produce a statistical estimate of the patient’s risk of having a fall [27,38] as well as a statistical estimate of their level of frailty [39]. QTUG has been shown to be reliable in the measurement of gait and mobility [40], as well as accurate in predicting falls in PD [22] and community dwelling older adults [38,41]. We believe a statistical model based on inertial sensor measures of movement has the potential to be used as a surrogate measure of falls counts in patients with PD.

The current literature supports the utility of an array of objective gait and mobility parameters as predictors of falls risk in PD patients; however, in clinical trials, self-reporting of the *falls count* is still considered the gold standard to evaluate the efficacy of new therapies. To the best of our knowledge, this is the first study employing comprehensive wearable sensor data to develop an algorithm to predict the number of falls that will be experienced by a person with PD. Our work will offer a preliminary clinical validation of a novel digital biomarker that can support the effect detection and interpretation of clinical trial outcomes. Given that falls can be catastrophic for people with PD, therapies and interventions that aim to reduce falls could have a significant and beneficial effect on the quality of life for disease sufferers.

## 2. Datasets

Three datasets containing a total of 1057 participants were included in the analysis, including 71 participants previously diagnosed with PD. The data consisted of one set of healthy community dwelling older adults recruited into a large research study (Technology Research for Independent Living “TRIL” dataset), referred to hereafter as the Training Dataset “TD”, and two sets of Parkinson’s Disease patients (Order of Saint Francis “PD1” dataset and University College Dublin’s “Healthy PD”, referred to as “PD2” dataset hereafter). All the participants completed at least one QTUG assessment depending on the study protocol. Table 1 below provides summary details on the three datasets included. Further details on each dataset are provided in the sections below and in Appendix A. Of the two PD datasets reported, the PD2 participants were considered less impaired than the PD1 participants (see UPRDS (Unified Parkinson’s Disease Rating scale part III) scores in Table 1).

Each study received ethical approval from the local ethics committee (see detailed information below). All the data reported here were anonymized and stored in line with data privacy regulations in each country (e.g., HIPAA, GDPR).

**Figure 1:**
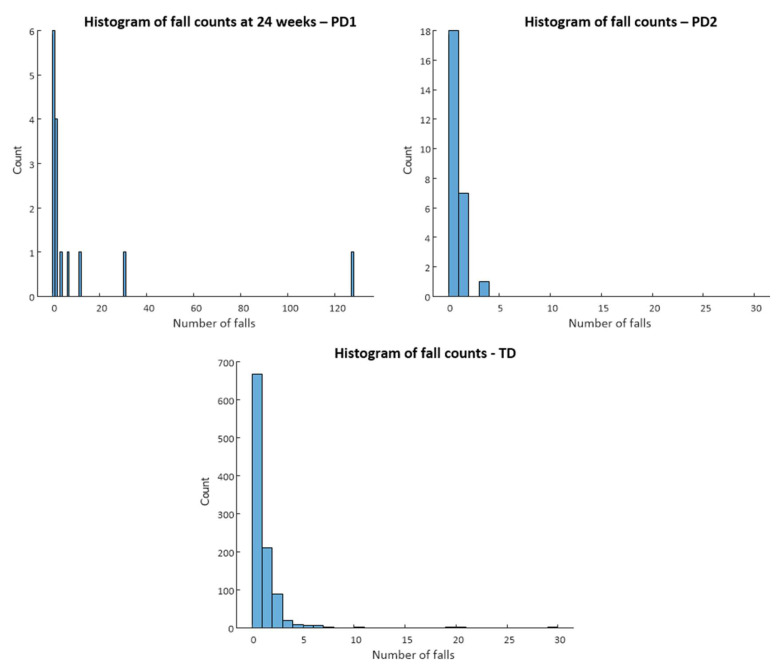
Histogram of falls counts for each dataset. Distributions are clustered around low numbers of falls, with a majority of participants in each dataset reporting no falls.

Marked differences in falls rates can be observed across the datasets, with the PD2 dataset reporting a rate of 0.37 falls per participant (measured retrospectively) in contrast to the PD1 data, which reported 12.1 falls per participant over the study duration (24 weeks measured longitudinally). The distribution of falls for each dataset is reported in Figure 1, which provides a histogram of the falls counts for each dataset. Falls distributions are clustered around low numbers of falls, with a majority (>50%) of participants in each dataset reporting no falls. The falls data obtained in TD were reported clinically to an experienced research nurse who cross-checked against hospital records (where possible). The PD1 falls data were obtained prospectively through daily falls diaries and collected weekly for 6 months from the baseline. The PD2 falls data were self-reported to the researcher at the time of each QTUG assessment over the 12-week study duration. The differences between the falls outcome data obtained for PD1 and PD2 meant that the two datasets could not be pooled and needed to be analyzed separately.

### 2.1. Training Dataset (TD)

The data used to train all the statistical models (Training Dataset (TD)) were obtained from the TRIL research project, which examined technologies to support positive ageing and included a focus on the prevention of falls. These data were combined with a number of other smaller datasets arising from separate research studies to form a reference dataset, used to train the falls risk estimate (FRE) classifier models and mobility risk scores included in the Kinesis QTUG™ product [27,38,42,43].

The data consisted mainly of community dwelling older adults assessed at St James Hospital, Dublin, Ireland and included N = 1015 subjects for analysis. Ethical approval was received from the St James hospital research ethics committee. Each participant completed a battery of functional tests, including the Timed Up and Go and a 6-meter walk, instrumented with inertial sensors. In addition, each participant received a Comprehensive Geriatric Assessment (CGA), which included vision tests, a medication review and a blood pressure and cardiovascular assessment. Participants also received a cognitive function assessment: the Mini Mental State Examination (MMSE). Participants had an average age of 72.2 years, mean height of 166.6 cm and mean weight of: 74.6 kg. Twenty-nine participants reported they had been diagnosed with PD prior to assessment.

**Inclusion criteria:** inclusion criteria were subjects 60 years and older, with no history of stroke, able to walk without assistance, able to provide written informed consent.

**Exclusion criteria:** aged under 60 years of age, unable to provide informed consent or MMSE less than 18.

### 2.2. Parkinson’s Disease Dataset 1 (PD1)

This study was a single site longitudinal study of Parkinson’s disease patients. A total of 16 participants were recruited from the OSF HealthCare-Illinois Neurological Institute (Peoria, IL, USA); sensor data were not available for one participant, leaving 15 participants for analysis (5 female, mean age 67.3 ± 7.1 years). Patients were assessed over a 6-month period; QTUG assessments were conducted on a monthly basis following an initial baseline assessment. A total of 94 QTUG recordings were available for the 15 participants. Participants were evaluated three times using the UPDRS part III: at baseline, 90 days and 180 days. The study [22] included a weekly falls diary as well as SF−36, UPDRS and medication information for each assessment. ‘ON’ or ‘OFF’ state was documented only as a function of the clinical data captured and has not been explicitly analyzed. Participants did not receive any pharmaceutical or other intervention over the course of the trial.

All patients were required to provide informed consent. Ethical approval was received from the Peoria Institutional Review Board.

**Inclusion criteria:** able to provide written informed consent, aged 40 to 80, Idiopathic Parkinson’s disease (meeting UK Brain Bank criteria), responsive to Levodopa for at least four years, MMSE score greater than 22 and able to walk at least 3 m independently.

**Exclusion criteria:** atypical Parkinsonism, Hoehn and Yahr stage V, MMSE 21 or less, use of assisted device for ambulation, co-morbidities affecting balance: severe neuropathy, weakness, bilateral hip replacement, syncopal episodes causing falls, diagnosed with lumbar radiculopathy, spinal stenosis or any other back conditions with the potential to affect fall behavior, drug abuse or alcoholism.

### 2.3. Parkinson’s Disease Dataset 2 (PD2)

The second Parkinson’s Disease dataset (PD2) arose from the “Healthy PD” study, conducted in University College Dublin (Dublin, Ireland), and examined the effect of a 12-week exercise intervention study [44] in patients with PD.

Twenty-seven subjects, each with idiopathic PD ranging from stage I to stage III on the Hoehn and Yahr (H&Y) staging scale, voluntarily consented to participate in the study. Experimental protocols were approved by the Human Research Ethics Committee for Sciences at University College Dublin. Participants were evaluated four times during the study. Following an initial baseline assessment, each participant took part in an exercise intervention program, was subsequently re-assessed post-intervention and then followed-up three months after study completion. The participants did not receive any pharmaceutical intervention over the course of the trial. The participants reported their history of falls in the previous 12 months at each QTUG assessment; one participant did not provide falls history.

**Inclusion criteria:** diagnosed with Parkinson’s, stage I to stage III on the Hoehn and Yahr (H&Y), able to provide written informed consent.

**Exclusion criteria:** none.

## 3. Methods

### QTUG Assessment Protocol

**Figure 2:**
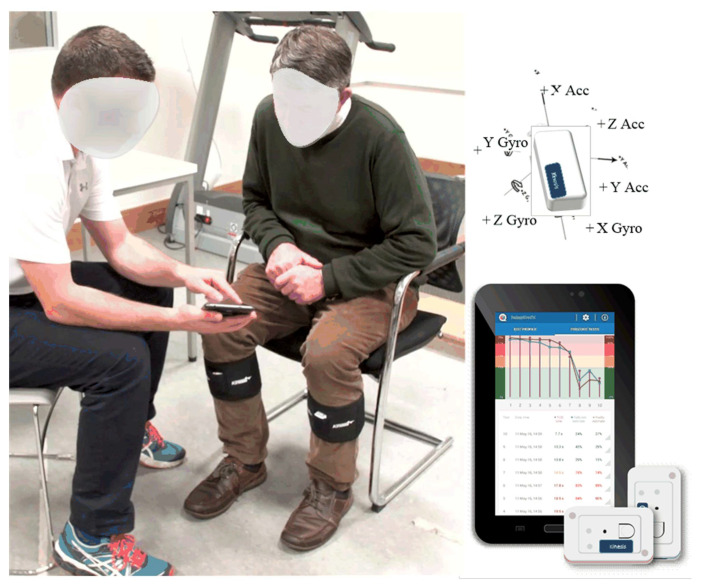
Inertial sensor is placed on the shin using a Velcro strap for each assessment. Sensor data are streamed to a tablet device via Bluetooth. Sensor accelerometer and gyroscope axes are shown.

Each participant was assessed using a Timed Up and Go (TUG) test, instrumented with inertial sensors (QTUG), placed on each shin below the knee (see Figure 2). QTUG assessments follow a highly prescriptive protocol: test distance measured as exactly 3 meters, turn point marked on the ground using tape (and not marked using a cone). Participants were always instructed to wear comfortable walking shoes and to complete the TUG “*as fast as safely possible*”. Every effort was made to control underfoot conditions (e.g., removing obstacles or loose carpeting) and to ensure at least four-meter linear space, ensuring adequate space to turn. Where possible, participants were encouraged to complete the test without the use of a walking aid; if a walking was used, this was noted in the software.

Each QTUG assessment (QTUG™, Kinesis Health Technologies, Dublin, Ireland) produces 71 different calculated parameters, which include a range of features quantifying gait, mobility, turning and transfers [27] (details on the processing applied to produce each parameter are reported elsewhere [38,45]). In addition, each assessment produces a statistical falls risk estimate (FRE_sensor_) [38,45] based on the inertial sensor data as well as a statistical Frailty estimate based on the inertial sensor data (FE_sensor_) [39].

## 4. Statistical analysis

### 4.1. Exploratory Analysis

The association of FRE_sensor_ produced by QTUG with falls count was explored for each dataset. A one-way ANOVA with significance level set to *p* < 0.05, where the number of falls is treated as an ordinal variable, was used to examine this relationship. As the TD dataset was used to develop and validate the current FRE_sensor_, only the PD1 and PD1 datasets were included in this analysis as they are statistically independent from the data used to generate FRE_sensor_.

The association of the mobility risk scores with falls counts is examined for the PD2 dataset, which is statistically independent of the reference dataset used in creating the mobility risk scores. A one-way ANOVA (with significance level set to *p* < 0.05), where falls count is treated as a categorical variable, was used to examine this relationship. 

The exploratory analysis of the association of falls counts with individual gait and mobility parameters produced by QTUG is included in Appendix B.

### 4.2. Predictive Model of Falls Counts

Two main approaches were taken to develop a novel method to predict fall rate (counts) in PD:

1. Using existing trained classifiers to predict falls counts (QTUG FRE and Mobility score models)
2. Ensemble model based on elastic net models with Poisson regression

For each approach, training and validation were carried out using the training data (TD) set, while testing on each of the models was carried out on two independent PD datasets (PD1 and PD2).

Falls count data for all models were log transformed to reduce the effect of zero-inflation on the distribution using the following expression:

LogNumFalls=log(1+NumFalls−min(NumFalls))

where min(*NumFalls*) is zero for all datasets reported here. To analyze or plot predicted falls counts against actual falls, the prediction can be converted back to *NumFalls* using the following expression:

NumFalls=exp(logNumFalls)−1+min(NumFalls)

All analyses were performed using Matlab v9.3 (R2017b).

#### 4.2.1. Predicting Falls Counts Using Existing QTUG Risk Estimates (FRE Model)

Two statistically independent datasets (PD1 and PD2) were used to test the performance of a predictive model of falls counts (referred to hereafter as the FRE model) based on measures produced by existing trained QTUG classifier models using three standard features: FRE_sensor_, FE_sensor_ and TUG time.

FRE_sensor_ and FE_sensor_ were trained using the training dataset, as reported elsewhere [38,39], and are based on regularized discriminant and logistic regression models, respectively. TUG time (the time to complete the TUG test) has been frequently shown in the literature to be associated with falls [46,47].

To test the performance of the FRE model, data for each of the independent PD datasets were then applied to the model using negative binomial regression. Negative binomial regression was used due to zero-inflation and over-dispersion of the falls count data. 

#### 4.2.2. Predicting Falls Counts Using QTUG Mobility Risk Scores (Mobility Score Model)

QTUG produces five mobility risk scores that were calculated from the reference dataset (N = 1495) [27,43] (this set contains data from both the TD and PD1 datasets, and a number of other clinical datasets). 

The PD2 dataset was used to examine the performance of the QTUG mobility risk scores in predicting falls count (Mobility score model). To test the performance of the Mobility score model, data for each of the independent PD datasets were then fitted to each independent dataset using negative binomial regression.

#### 4.2.3. Predicting Falls Counts with Elastic Net Regression

To train and validate a novel predictive model for falls counts in PD using QTUG data, a cross-validated elastic net procedure with a Poisson distribution was used. The model was trained using the training dataset and tested on the two independent PD datasets (PD1 and PD2).

The training data were considered in a number of different subsets as follows:

- TD-All—All training data
- TD-Fallers—training data excluding non-fallers (number of falls >0)
- TD-PD—PD patients within the training dataset only
- TD-Fallers-PD—PD patients who had experienced at least one fall
- TD-NoPD—training dataset excluding PD patients
- TD-Fallers-NoPD—dataset excluding fallers and patients with PD

A set of trained regression models was produced for each of the training datasets listed above. To produce a trained model for testing on the independent datasets, the selected model was trained on all available training data and applied to the test set. Where validation was required prior to testing, the training set was split into training and validation sets. In all approaches, model selection was performed using 10-fold cross-validation to reduce bias.

For the elastic net model, an alpha value was set, a priori, to 0.1; models were also a priori constrained to a minimum model size of three and a maximum model size of 20. Model selection was also constrained to include the TUG time and to exclude a number of features with previously reported poor reliability (such as stance time asymmetry).

We considered an ensemble of regression models where the ensemble prediction is completed by predicting falls count as a linear combination of the estimates from the TD-NoPD and TD-Fallers-NoPD models. The ensemble coefficients for the linear combination were obtained through linear regression, estimating true falls count using predictions from each constituent model on the TD-PD validation set.

The PD1 dataset contains a number of very large outlier falls count values (e.g., *NumFalls* = 127) that distort model predictions. The results are presented for all the data as well as with outliers removed (*NumFalls* > 10). For the training set, a small number of outliers were removed (where *NumFalls* > 10). The PD2 dataset did not contain any outliers.

#### 4.2.4. Model Performance Metrics

The performances of each model on the training and testing sets were evaluated using the following metrics: coefficient of determination (R^2^), root mean squared error (RMSE), Spearman’s rank correlation (ρ) and model size (number of features).

The coefficient of determination was included for completeness as a measure of ‘goodness of fit’. However, as the falls count data approximate a zero-inflated Poisson distribution, it should be noted that this measure does not always provide a reliable assessment of model fit in the presence of outliers or with low samples sizes.

## 5. Results

We report the results for a range of statistical models intended to predict the fall rates in PD patients. The sensor data for all the datasets were not normally distributed. Falls count data were heavily clustered around zero, suggesting that the data may follow a negative binomial or a zero-inflated Poisson distribution.

The results of a battery of statistical tests analyzing the association with falls counts for TD, PD1 and PD2 datasets are included in Appendix B.

### 5.1. Predictive Model of Falls Counts Using QTUG

The results for the predictive model of falls counts using QTUG data are detailed below. This involved training and validating a suite of models using the training (TD) dataset. The selected models were then tested on the two independent PD datasets, which were held out from all the model training.

#### 5.1.1. Existing QTUG Falls Risk Model

**Figure 3:**
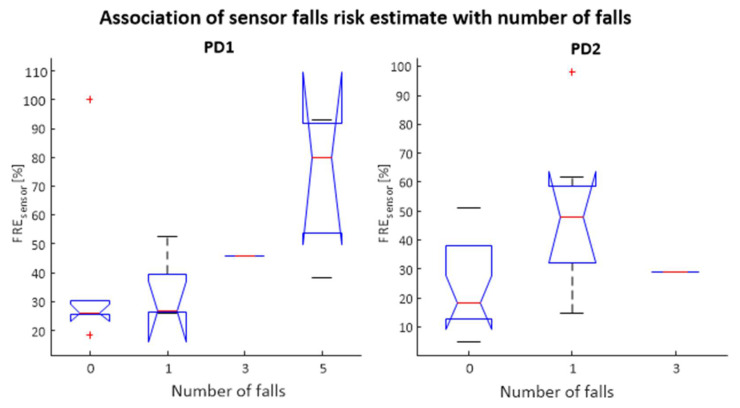
Association between FRE sensor and falls counts for two statistically independent datasets (PD1 (**left**) and PD2 (**right**)). The PD1 dataset took falls counts at 24 weeks post baseline assessment.

This section details the performance of the previously trained QTUG FRE_sensor_ digital biomarker as a surrogate measure of falls counts as well as in classifying falls risk. A significant association between FRE_sensor_ and falls count was observed for the PD2 dataset (F = 4.37, *p* < 0.05, respectively). The PD2 dataset showed a notable but non-significant association (F = 2.01, *p* = 0.17) with falls counts. Figure 3 shows the association of FRE_sensor_ with falls counts for each of the PD1 and PD2 datasets.

The results are reported in Table 2 for the prediction of falls counts using an existing pre-trained QTUG classifier model (FRE model) tested on two independent datasets (PD1 and PD2). A mean RMSE of 0.42 and a mean Spearman’s correlation coefficient of 0.30 were obtained when the model performance was averaged across two independent PD datasets.

The PD1 dataset (N = 15) contained a number of very large outlier falls count values (e.g., Number of falls (*NumFalls*) = 127), which distort model predictions. The results are presented for all the data as well as with outliers removed (Number of Falls >10). In addition, we present the data when grouped into falls count categories (where Number of falls >5 is placed into the five falls categories); the results are provided for three different scenarios (tested on all data, outliers removed and placed into categories 0–5+).

The PD2 data (N = 26) did not contain any outliers, so the results with outliers excluded are not presented. For the training set, outliers were also removed.

In addition, a negative binomial model was fit to the mobility scores (Mobility model) and tested using the PD2 dataset, which yielded ab RMSE of 0.33 and correlation coefficient of 0.55.

**Figure 4:**
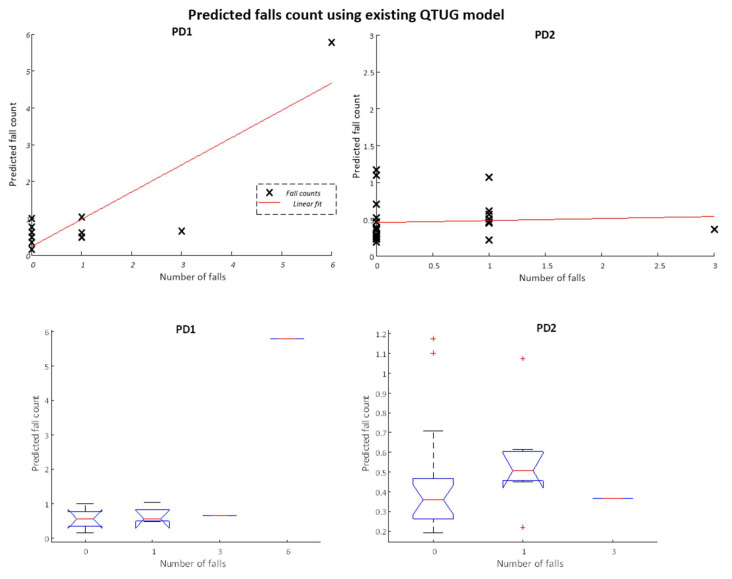
Predicted falls count versus actual number of falls for existing QTUG data model tested on independent PD test datasets (PD1 (**left**) and PD2 (**right**)). For PD1 dataset, outliers (Number of Falls >10) are removed, for PD2 data set the maximum fall count for any participant was 3. Top panels plot actual fall counts against predicted while bottom panels group fall counts into buckets (0–6).

Figure 4 below shows a scatter plot (top panel) and boxplots (bottom panel) of predicted falls counts versus actual falls counts for the existing QTUG data model, tested on the two independent datasets. For the PD1 data, outliers where number of falls is greater than 10 are removed.

#### 5.1.2. Elastic Net Ensemble Models

##### Training Falls Count Models Using Cross-Validation

The model selection was conducted using 10-fold cross-validation for each model. Once the model selection was complete, all the training data from each subset were fitted to the data by re-substitution in order to produce a set of coefficients for each model, along with results for the performance of the model on the training data. The training results and coefficients for each elastic net regression model and sub-set are detailed in Appendix C and Appendix D respectively.

##### Testing Falls Count Models on Independent PD Datasets

The performance of each of the trained elastic net falls count models on each of the independent Parkinson’s disease tests sets is detailed in Table 3 below. The ‘TD-All’ model yielded a mean RMSE of 0.62 and Spearman’s rank correlation coefficient of 0.27 across the two statistically independent PD datasets.

**Figure 5:**
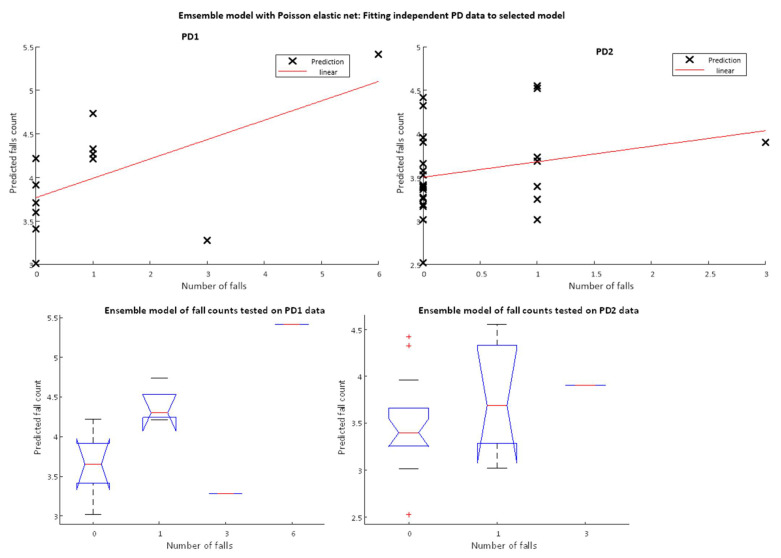
Testing ensemble model on independent PD datasets. Ensemble model is trained using TD-NoPD and TD-Faller-No-PD datasets and validated on TD-PD dataset.

Figure 5 below demonstrates the performance of the ensemble model when tested on the two independent PD datasets as both a scatter plot and a boxplot. The results show a mean RMSE of 1.28 and mean rank correlation coefficient of 0.38. 

## 6. Discussion

This manuscript reports the results of a wearable sensor-based method to generate surrogate measures of falls counts in PD. We believe this approach has the potential to be further developed as a more sensitive readout of falls in PD. Three distinct and independent datasets, containing a total of 1057 participants (including 71 previously diagnosed with PD) were included in the analysis.

Using a comprehensive kinematic assessment, we explored the possibility to predict falls counts using two existing trained models (the FRE model and Mobility model), previously tested for falls risk assessment, and a novel mathematical approach using elastic net, ensemble learning and Poisson regression. Previous research has shown that the FRE and Mobility score models were associated with falls in a number of populations. The gait and mobility parameters produced by the QTUG algorithm can be noisy and mutually correlated, so the feature vector dimension needs to be reduced through feature or model selection. This point, combined with the fact that falls counts are thought to follow a zero-inflated Poisson process, meant that we considered an elastic net procedure combined with Poisson regression and ensemble modelling to be a promising means to obtain an accurate model of falls counts.

The results for the FRE model found that falls counts can be predicted with a mean RMSE of 0.42 and a mean correlation of 30% with falls counts for two statistically independent datasets of patients with PD. Similarly, the results for the Mobility model found that falls can be predicted with an RMSE of 0.33 and correlation coefficient of 0.55 when tested on an independent dataset of PD patients. An ensemble of Poisson regression models produced a mean RMSE of 1.28 and mean rank correlation coefficient of 0.38, while the results, averaged across a separate male/female elastic net Poisson regression model, yielded an RMSE of 0.57 and correlation with falls counts of 0.29 for two independent datasets. The best results reported here were obtained by using an existing trained classifier model (FRE model) to predict falls counts. However, some limitations should be considered in the interpretation of our findings. First, we believe the ensemble modelling approach was hampered by a small sample of PD patients (given the FRE model was trained on a much larger, more varied dataset) and may be more promising and might perform better when trained with a larger and more representative dataset containing a larger proportion of PD patients. Second, the falls count data for PD1 contained a number of extreme outliers and, to evaluate their effects, the analyses were performed both including and excluding those samples. As expected, outliers had a significant impact on the reported outcome, probably due to the fact that the training data (TD) only contained low falls counts and, as a result, the model did not generalize well to extremely large falls count values. Third, due to the small sample size, medication status, on/off periods and disease severity were not considered in the analysis. Furthermore, the PD2 dataset was perhaps unusually healthy for a sample of PD patients, able to undertake a 12-week exercise program (with no participants dropping out) that consisted of spinning, circuit training and tai-chi; this may have led to lower than expected numbers of falls. Moreover, it is important to consider the intrinsic nature of falls, which are essentially a random, stochastic event. For this reason, predicting the exact number of falls that will occur in a given time frame is inherently difficult; the infrequency of the event belies the risk that may or may not be captured within the clinical trial horizon. However, this approach, in combination with statistical fall risk estimates, could provide a clinical view with a higher level of granularity; statistical indices, which produce a probabilistic estimate of future falls based on an analysis of movement, might offer additional insight into the state of the neuromuscular control system as opposed to solely relying on an approach that aims to catch an infrequent and potentially catastrophic endpoint (i.e., a fall event).

The results also suggest a significant association between the number of falls (falls counts) and the sensor-based falls risk estimate model (FRE_sensor_) for community dwelling older adults reported previously [22,27] and currently deployed in a commercial product (Kinesis QTUG™). In addition, strong associations were observed between falls counts and a number of individual gait and mobility parameters, particularly measures of gait variability and average values of temporal–spatial gait during the TUG test (Appendix B).

Several studies have examined the value of instrumented gait and mobility tests in the assessment of Parkinson’s [22,29,48,49]. A recent meta-analysis of 26 studies [50] found that spatiotemporal characteristics of gait, such as slower walking speed, lower cadence and shorter strides, can increase the risk of future falls. Importantly, clinical features can be combined with spatiotemporal gait dynamics to elucidate falls pathophysiology [51]. The most consistent results are in relation to stride time variability, which was significantly associated with falls counts (fall frequency) and not related to tremor, rigidity or bradykinesia in the “off” state [52]. However, stride time variability significantly improved in response to levodopa, both in fallers and non-fallers, but remained increased in fallers when compared to non-fallers. Hoskovcová et al. [34] found that stride time variability may predict falls in prospectively identified PD fallers, which agrees with previous research by Hausdorff et al. [53] and Lord et al. [54] suggesting the increased gait variability predicts falls in community dwelling older adults [55]. In addition, authors found that stride time variability correlated with the total BDI-II score, which was increased in PD fallers. While the association of various measures of gait with falls in PD has been well-established, this study demonstrates the potential of combining such measures into a predictive model for use as a clinical trial endpoint.

A limitation of this study is the small sample sizes available for the two independent PD datasets; as such, the results reported here may need to be replicated in a larger study.

Future work will aim to replicate these findings in a larger study, including the evaluation of longitudinal relationships between mobility parameters, falls counts and UPDRS scores in PD, and their utility in measuring disease progression. Improvements in the predictive model for falls counts will entail an extended ensemble model approach that would include both the FRE and Mobility score models.

## 7. Conclusions

To conclude, our findings support the goal of integrating wearable sensor technology into the clinical and routine care of patients with movement disorders and may offer novel objective endpoints for future clinical trials.

## Figures and Tables

**Table 1:** Summary details per dataset. MMSE refers to Mini Mental State Examination. CGA refers to Comprehensive Geriatric Assessment, includes vision, blood pressure and medication review. BMI refers to Body Mass Index. UPDRS refers to Unified Parkinson’s Disease Rating scale (UPDRS) part III. Falls rate is mean falls per patient. TUG time is the mean time to complete the TUG test, while gait velocity is mean stride velocity during the TUG. Falls rate is reported as the mean number of falls per patient over the study window.

Dataset	TD	PD1	PD2
N (M/F)	1015 (344/671)	15 (10/5)	27 (17/9)
Population	Community dwelling Control Residential care	Parkinson’s disease	Parkinson’s disease
Study type	Cross-sectional Longitudinal	Longitudinal	Cross-sectional
Outcomes	Clinically reported falls CGA MMSE	Weekly falls diaries UPRDS	Self-reported falls UPDRS
UPDRS part III	-	15.1 ± 9.6	22.56 ± 10.25
Fallers/Non-fallers	409/606	4/11 (12 weeks) 8/7 (24 weeks)	8/18
Total falls (Falls rate)	652 (0.64)	181 (12.1)	10 (0.37)
TUG time (s)	10.8 ± 3.9	12.5 ± 4.3	8.6 ± 2.9
Gait velocity (cm/s)	98.9 ± 18.7	89.4 ± 24.5	116.0 ± 14.9
Age (yrs)	72.2 ± 10.9	67.3 ± 7.1	64.9 ± 7.3
Height (cm)	166.6 ± 9.8	172.9 ± 9.8	171.2 ± 8.3
Weight (kg)	74.6 ± 16.3	80.3 ± 15.7	74.7 ± 13.6
BMI	26.97 ± 4.70	26.86 ± 4.37	25.40 ± 3.79

**Table 2:** Testing of existing QTUG data model using negative binomial falls count model fit to independent PD datasets. Results are presented for both the FRE and Mobility models. For the PD1 dataset, results are provided for all data as well as with outliers (*NumFalls* > 10) removed.

Dataset	Model	N	R^2^	RMSE	Rho	#Features
PD1 (all data)	FRE	15	0.50	1.27	0.64	3
PD1 (outliers excluded)	FRE	12	0.73	0.41	0.44	3
PD1 (0–5+categories)	FRE	15	0.70	0.70	0.69	3
PD2 (all data)	FRE	26	0.13	0.42	0.15	3
PD2 (all data)	Mobility	26	0.48	0.33	0.55	5

**Table 3:** Results for Poisson elastic net model tested on independent PD datasets (PD1 and PD2). Results are provided for each of the models derived from the TD training subsets. Spuriously high values are indicated by ##. Outliers (*NumFalls* > 10) were excluded from analysis.

	PD1					PD2				
FRE	N	R^2^	RMSE	ρ	#Features	N	R^2^	RMSE	ρ	#Features
TD-All	12	0.00	0.80	0.38	13	26	0.00	0.45	0.16	13
TD-Fallers	12	0.00	0.62	0.34	13	26	0.00	0.38	0.28	13
TD-PD	12	0.00	##	−0.31	19	26	0.00	##	−0.05	19
TD-Fallers-PD	12	0.00	##	−0.38	4	26	0.00	##	−0.10	4
TD-NoPD	12	0.00	0.80	0.35	14	26	0.00	0.45	0.15	14
TD-Fallers-NoPD	12	0.00	0.73	0.52	20	26	0.00	0.41	0.24	20
Ensemble-TD-PD	12	0.00	1.23	0.52	2	26	0.00	1.36	0.24	2
**Male**										
TD-All	9	0.00	0.54	0.07	4	17	0.00	0.26	0.22	4
TD-Fallers	9	0.00	0.49	−0.45	6	17	0.00	0.39	0.47	6
TD-PD	9	0.00	18.81	−0.60	4	17	0.00	16.32	0.03	4
TD-Fallers-PD	9	0.00	12.77	−0.34	11	17	0.00	5.44	0.06	11
TD-NoPD	9	0.00	0.61	−0.07	20	17	0.00	0.29	−0.31	20
TD-Fallers-NoPD	9	0.00	0.51	−0.22	13	17	0.00	0.33	0.60	13
Ensemble-TD-PD	9	0.00	0.69	0.22	2	17	0.00	0.84	−0.60	2
**Female**										
TD-All	3	0.00	0.98	0.50	12	9	0.00	0.52	0.37	12
TD-Fallers	3	0.00	1.19	0.50	18	9	0.00	0.63	0.47	18
TD-PD	3	0.00	0.81	1.00	4	9	0.00	0.67	−0.21	4
TD-Fallers-PD	3	0.00	191.41	−1.00	20	9	0.00	##	0.02	20
TD-NoPD	3	0.00	0.92	0.50	10	9	0.00	0.48	0.58	10
TD-Fallers-NoPD	3	0.00	1.19	0.50	17	9	0.00	0.64	0.38	17
Ensemble-TD-PD	3	0.00	1.19	0.50	2	9	0.00	0.40	−0.34	2

**Figure A1:**
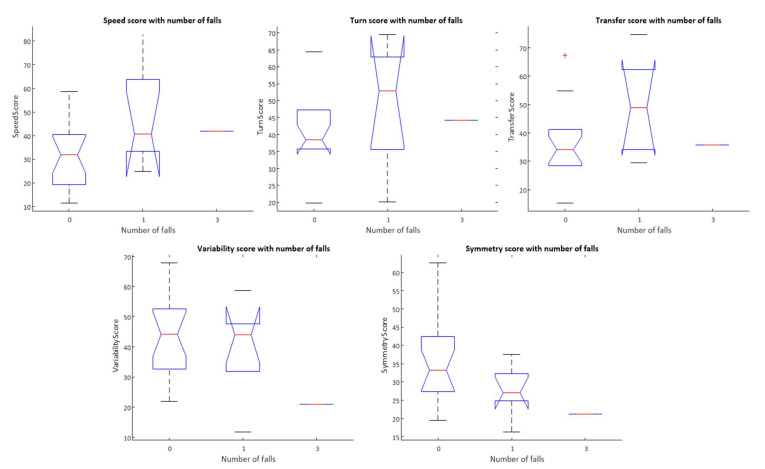
Association of mobility risk scores with number of falls for PD2 dataset. Boxplots for each mobility score are shown (Top row: Speed score, Turn score, Transfer score; Bottom row: Variability score, Symmetry score).

**Figure A2:**
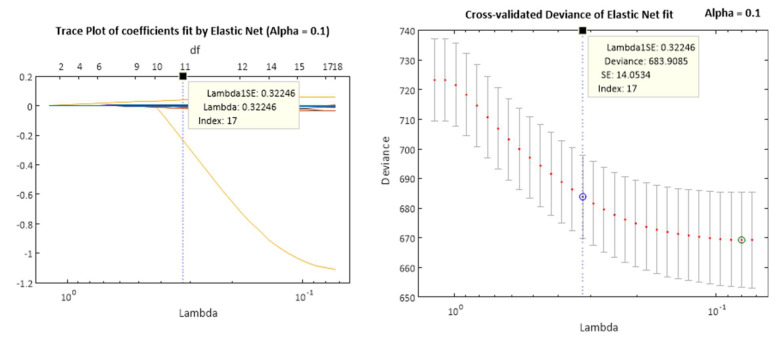
Elastic net trace and deviance plots for TD-All fit; lambda is selected as the value giving minimum deviance for a model size greater than or equal to 3 and less than or equal to 20.

**Figure A3:**
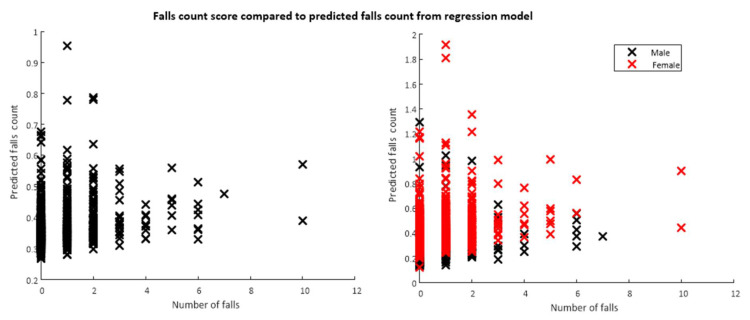
Poisson model fit with elastic net to TD training set data (TD-All model). Data are shown for both the all data and gender stratified (male/female) models.

## Data Availability

The data that support the findings of this study are available from the Principal Investigators of each of the original studies. Restrictions apply to the availability of these data, which were used under license for the current study, so are not publicly available. Data are, however, available from the authors upon reasonable request and with permission of the investigators.

## Abbreviations

Abbreviations used in this manuscript.

**Abbreviation**	**Definition**
PD	Parkinson’s Disease
*NumFalls*	Number of falls
QTUG	Quantitative Timed Up and Go
TUG time	Time to complete Timed Up and Go test
TD	Training dataset for elastic net Poisson regression models
PD1	Parkinson’s test dataset #1
PD2	Parkinson’s test dataset #2
UPDRS	Unified Parkinson’s Disease Rating Scale
MMSE	Mini Mental State Examination
CGA	Comprehensive Geriatric Assessment
FRE_sensor_	Inertial sensor-based estimate of falls risk
FE_sensor_	Inertial sensor-based estimate of frailty, based on Fried’s frailty phenotype
Mobility scores	Percentile-based scores quantifying mobility across five dimensions (Speed, Transfers, Turning, Variability, Symmetry), using inertial sensors, compared to a large reference dataset
FRE model	Negative binomial model of falls counts using TUG time FRE_sensor_ and FE_sensor_
Mobility model	Negative binomial model of falls counts using mobility scores

## Appendix A. Anthropomorphic Data

Anthropomorphic and clinical data for each dataset, stratified by gender, are included in Table A1 below.

**Table A1:** Anthropomorphic and clinical data separated by gender for each dataset.

	TD			PD1			PD2		
	All (N = 1015)	M (N = 344)	F (N = 671)	All (N = 15)	M (N = 10)	F (N = 5)	All (N = 27)	M (N = 17)	F (N = 9)
Age (yrs)	71.52 ± 11.34	69.65 ± 13.75	72.48 ± 9.75	67.29 ± 7.11	67.26 ± 7.06	67.33 ± 8.03	64.92 ± 7.28	64.88 ± 8.86	65.00 ± 2.92
Weight (kg)	74.03 ± 14.22	82.11 ± 11.89	69.88 ± 13.53	80.05 ± 15.64	85.81 ± 10.96	68.51 ± 18.33	74.73 ± 13.55	79.06 ± 11.54	66.56 ± 13.88
Height (cm)	165.61 ± 9.37	174.25 ± 7.27	161.17 ± 6.92	172.29 ± 9.77	176.94 ± 7.13	162.98 ± 7.56	171.24 ± 8.27	175.54 ± 6.24	163.11 ± 4.70
BMI	26.97 ± 4.70	27.05 ± 3.76	26.92 ± 5.11	26.86 ± 4.37	27.34 ± 2.20	25.91 ± 7.35	25.40 ± 3.79	25.63 ± 3.33	24.96 ± 4.74
TUG time (s)	10.77 ± 3.94	10.55 ± 3.52	10.88 ± 4.14	12.60 ± 2.47	11.77 ± 2.55	14.26 ± 1.23	8.60 ± 2.92	9.11 ± 3.38	7.62 ± 1.47
Mean velocity (cm/s)	98.90 ± 18.65	100.17 ± 19.81	98.25 ± 18.01	87.50 ± 10.08	88.52 ± 11.91	85.47 ± 5.33	116.03 ± 14.88	113.16 ± 16.10	121.45 ± 11.11

## Appendix B. Association of QTUG Parameters with Falls Counts

QTUG sensor parameters often do not follow perfectly normal distributions, so non-parametric statistical tests are used where appropriate. As the number of falls is a count variable, it can be modelled as an ordinal variable or a Poisson process.

Three sets of statistical tests were carried out for each dataset using binary falls classification (faller/non-faller) and falls count data (number of falls). The Mann–Whitney rank sum test was used to compare each QTUG parameter for discrimination between faller and non-faller populations, where a faller is defined as having one injurious fall or more than one previous fall. Spearman’s rank correlation was used to examine the relationship of each QTUG parameter against falls count data. One-way ANOVA, using the falls count as the categorical variable, was used to examine the association of the QTUG parameters with falls counts.

The alpha value for each hypothesis test was set to *p* < 0.05 to detect statistical significance. Where possible, falls counts were analyzed in the 0–5+ range, but data for each value in this range were not available for all datasets (e.g., PD2, only 0, 1, 3 counts available).

Note: the statistical tests detailed above should be considered as purely exploratory. Model selection is not performed based on this analysis due to the possibility of a type I error arising from multiple comparisons.

Seventy-one QTUG sensor parameters were included in the analysis per dataset [27,38]. These parameters can be grouped into categories as follows: falls risk and frailty scores, mobility risk scores, temporal gait parameters, spatial gait parameters, turn parameters, gait variability, gait symmetry, angular velocity parameters.

### Appendix B.1. Training Data (TD) Set

Table A2 details the results of a battery of statistical tests conducted on QTUG parameters calculated from the TD dataset (largely drawn from a sample of community dwelling older adults). Analyses examined statistical differences in each measure between fallers and non-fallers as well as the relationship between each measure and the number of falls reported by participants.

**Table A2:** Statistical analysis of QTUG parameters with falls counts for TD dataset. M and F refer to population stratified by gender (male and female). U refers to Mann–Whitney rank sum statistic, ρ refers to Spearman’s correlation coefficient, while F refers to F-score from 1-way Anova test. Statistically significant differences (*p* < 0.05) are indicated by *.

	Mann–Whitney			Spearman			Anova		
Parameter Name	Faller (Mean ± Std)	Non-Faller (Mean ± Std)	Rank Sum	ρ(All)	ρ(M)	ρ(F)	F(All)	F(M)	F(F)
Turn mid-point time (s)	5.22 ± 2.44	4.12 ± 1.35	245,646 *	0.24 *	0.21 *	0.23 *	14.76 *	5.31 *	10.04 *
Mean stride length (cm/s)	123.50 ± 22.03	133.67 ± 20.37	175,859 *	−0.22 *	−0.19 *	−0.20 *	12.55 *	3.64 *	7.68 *
TUG test time (s)	11.63 ± 4.83	10.19 ± 3.07	228,827 *	0.18 *	0.19 *	0.18 *	11.02 *	2.74 *	9.09 *
Number of gait cycles	6.56 ± 2.01	5.92 ± 1.49	232,287 *	0.19 *	0.17 *	0.18 *	10.71 *	2.96 *	7.19 *
Mean stride velocity (cm/s)	94.47 ± 19.24	101.89 ± 17.61	180,628 *	−0.19 *	−0.20 *	−0.18 *	10.19 *	3.31 *	7.17 *
Number of steps	13.60 ± 4.01	12.34 ± 2.97	232,030 *	0.18 *	0.16 *	0.16 *	10.17 *	2.90 *	6.78 *
Walk time (s)	9.05 ± 3.86	7.90 ± 3.01	232,167.5 *	0.19 *	0.19 *	0.19 *	8.99 *	2.75 *	6.64 *
Return from turn time (s)	5.27 ± 2.42	4.67 ± 2.16	229,265 *	0.18 *	0.22 *	0.16 *	6.63 *	2.41 *	4.56 *
Time to Sit (s)	1.83 ± 1.75	2.30 ± 1.42	188,460.5 *				6.07 *	2.38 *	5.15 *
CV *Z*-axis ang. vel. (%)	4.46 ± 1.18	4.55 ± 1.13	192,059 *	−0.11 *	−0.16 *	−0.11 *	5.46 *	6.89 *	2.95 *
CV *X*-axis ang. vel. (%)	4.47 ± 1.18	4.56 ± 1.14	194,051 *	−0.10 *	−0.15 *	−0.09 *	5.36 *	6.90 *	2.96 *
CV *Y*-axis ang. vel. (%)	4.44 ± 1.18	4.50 ± 1.12	202,533	−0.04	−0.08	−0.03	4.97 *	6.36 *	2.80 *
Number of strides in turn	2.57 ± 0.99	2.35 ± 0.82	222,415.5 *	0.12 *	0.13 *	0.09 *	4.93 *	1.47	3.56 *
Min *Y*-axis ang. vel. × Height (deg·m/s)	−369.35 ± 86.63	−380.76 ± 85.97	215,481	0.09 *	0.06	0.10 *	4.77 *	1.25	4.07 *
Min *Z*-axis ang. vel. x Height (deg·m/s)	−292.71 ± 113.61	−330.12 ± 127.30	230,349 *	0.16 *	0.05	0.17 *	4.44 *	0.12	4.84 *
CV stride velocity (%)	3.51 ± 0.93	3.51 ± 0.86	214,948	0.00	0.02	0.00	4.41 *	6.18 *	2.90 *
Magnitude mean at mid-swing points (deg/s)	276.85 ± 60.13	282.06 ± 50.28	202,768	−0.06 *	−0.07	−0.10 *	4.21 *	1.47	3.53 *
Max *Y*-axis ang. vel. × Height (deg·m/s)	602.28 ± 112.62	613.78 ± 111.34	201,741	−0.08 *	−0.05	−0.09 *	3.99 *	0.99	3.64 *
Min *Y*-axis ang. vel. (deg/s)	−226.12 ± 53.33	−228.01 ± 49.32	209,191	0.05	0.03	0.08 *	3.90 *	1.12	3.30 *
Turning time (s)	3.20 ± 1.54	2.86 ± 1.55	225,713 *	0.15 *	0.18	0.13 *	3.82 *	1.24	3.17 *
Min *Z*-axis ang. vel. (deg/s)	−178.46 ± 67.06	−197.32 ± 74.50	227,152 *	0.13 *	0.04	0.17 *	3.49 *	0.10	4.34 *
Mean *X*-axis ang. vel. (deg/s)	47.61 ± 18.02	47.69 ± 17.86	207,329	−0.02	−0.01	−0.04	3.19 *	0.83	3.07 *
Mean single support	0.39 ± 0.05	0.40 ± 0.05	192,517 *	−0.11 *	−0.08	−0.11	3.14 *	1.17	2.29 *
Cadence (steps/min)	94.55 ± 15.54	97.11 ± 14.24	198,692 *	−0.09 *	−0.11	−0.12	3.13 *	1.70	3.05 *
Min *X*-axis ang. vel. × Height (deg·m/s)	−705.10 ± 213.10	−740.84 ± 206.64	219,102 *	0.07 *	0.04	0.09	3.12 *	0.93	3.08 *
CV stride length (%)	3.09 ± 0.85	3.02 ± 0.81	214,948	−0.01	0.00	0.01	3.10 *	3.45 *	2.54 *
Mean *X*-axis ang. vel. × Height (deg·m/s)	77.82 ± 29.63	79.65 ± 30.35	203,230	−0.04	−0.02	−0.05	3.07 *	0.77	3.17 *
Max *Y*-axis ang. vel. (deg/s)	368.67 ± 68.29	367.82 ± 64.97	210,123	−0.02	−0.02	−0.06	3.00 *	0.83	2.68 *
Time to stand (s)	1.60 ± 1.52	1.17 ± 1.01	157,907.5 *				2.99 *	2.65 *	2.01
Mean double support	0.23 ± 0.09	0.22 ± 0.07	220,289 *	0.10 *	0.10	0.10	2.88 *	0.93	2.53
Single support variability (%)	2.48 ± 0.81	2.41 ± 0.80	215,286	−0.01	0.00	−0.02	2.83 *	2.10	2.50 *
Max *Z*-axis ang. vel. (deg/s)	228.29 ± 78.44	219.95 ± 77.33	215,623	0.00	0.00	−0.01	2.75 *	2.28 *	2.13
Min *X*-axis ang. vel. (deg/s)	−431.13 ± 127.99	−444.08 ± 122.74	214,101	0.04	0.02	0.08 *	2.67 *	0.83	2.64 *
Double support variability (%)	3.40 ± 0.96	3.37 ± 0.94	212,048	−0.02	−0.04	0.00	2.61 *	3.69 *	1.89
Max *Z*-axis ang. vel. x Height (deg·m/s)	373.75 ± 131.33	367.73 ± 133.24	211,979	−0.03	−0.01	−0.02	2.61 *	2.27 *	2.35 *
Mean *Y*-axis ang. vel. × Height (deg·m/s)	94.53 ± 33.82	96.87 ± 34.56	204,249	−0.05	−0.04	−0.05	2.44 *	0.76	2.45 *
Max *X*-axis ang. vel. × Height (deg·m/s)	697.81 ± 229.74	737.93 ± 223.70	194,870 *	−0.09 *	−0.08	−0.11 *	2.39 *	0.46	2.30 *
Swing time variability (%)	2.59 ± 0.88	2.57 ± 0.84	209,418	−0.03	−0.02	−0.04	2.37 *	3.24	1.69
Mean stance time (s)	0.81 ± 0.18	0.78 ± 0.16	216,963 *	0.08 *	0.10	0.09 *	2.27 *	1.31	2.34 *
Mean *Y*-axis ang. vel. (deg/s)	57.90 ± 20.70	58.05 ± 20.50	208,277	−0.02	−0.03	−0.03	2.18	0.76	2.18 *
Stride length asymmetry (%)	2.09 ± 32.69	2.70 ± 20.31	194,835				2.07	1.28	1.24
Stride velocity asymmetry (%)	1.85 ± 32.40	3.68 ± 17.72	209,999		0.06		1.87	1.24	1.20
Mean swing time (s)	0.49 ± 0.07	0.50 ± 0.06	195,354 *	−0.07 *	0.01	−0.05	1.78	0.32	1.91
Max *X*-axis ang. vel. (deg/s)	427.09 ± 140.18	442.08 ± 130.96	198,781 *	−0.07 *	−0.07	−0.09 *	1.77	0.33	1.94
Mean *Z*-axis ang. vel. (deg/s)	28.22 ± 12.35	26.72 ± 11.02	216,017	0.01	0.08	0.00	1.71	1.93	2.03
Mean stride time (s)	1.30 ± 0.20	1.28 ± 0.18	212,414.5	0.05	0.10	0.07	1.67	1.41	1.79
Stance time variability (%)	3.29 ± 0.99	3.23 ± 0.99	213,340	−0.01	0.01	0.00	1.58	1.98	1.43
Mean *Z*-axis ang. vel. x Height (deg·m/s)	46.29 ± 20.84	44.75 ± 18.99	212,131	−0.01	0.07	−0.01	1.45	1.65	2.16
Stride time variability (%)	2.90 ± 0.93	2.79 ± 0.92	217,195 *	0.01	0.03	0.01	1.42	2.19	1.53
Walk ratio	1.08 ± 0.39	1.17 ± 0.52	197,637 *	−0.02	0.03	−0.03	1.40	2.92 *	0.96
Step time asymmetry (%)	1.09 ± 25.88	0.32 ± 22.55	206,480	0.06 *	0.00	0.09 *	1.31	1.43	1.47
Mean step time (s)	0.61 ± 0.13	0.60 ± 0.09	206,948	0.02	0.00	0.06	1.25	0.38	1.56
Step time variability (%)	2.69 ± 0.96	2.61 ± 0.88	214,216	−0.01	−0.05	0.01	1.17	2.11	1.15
Turn magnitude (deg/s)	87.47 ± 83.66	87.56 ± 86.78	208,458	0.02	−0.08	0.06	1.14	1.08	1.10
Swing time asymmetry (%)	−1.16 ± 18.25	−1.42 ± 14.54	208,523	0.02	0.03	0.01	0.98	0.36	0.84
Stride time asymmetry (%)	0.62 ± 17.91	−1.96 ± 15.32	216,223.5	0.04	0.09	0.03	0.83	1.16	0.33
Stance time asymmetry (%)	1.23 ± 27.91	−1.77 ± 24.76	213,976.5	0.02	0.08	0.01	0.81	0.78	0.67
Magnitude range at mid-swing points (deg/s)	225.93 ± 64.89	222.08 ± 66.36	212,668	0.01	−0.02	0.00	0.62	0.68	0.63
Ratio strides/turning time	0.88 ± 0.35	0.88 ± 0.30	204,135.5	−0.04	−0.04	−0.07	0.53	0.51	0.51

### Appendix B.2. PD1 Dataset

Table A3 details the results of a battery of statistical tests conducted on QTUG parameters calculated from the PD1 dataset (longitudinal study of Parkinson’s Disease patients) [22]. Analyses examined statistical differences in each measure between fallers and non-fallers as well as the relationship between each measure and the number of falls reported by participants. Strong correlations were observed between a number of gait variability and temporal–spatial gait measures and falls counts.

**Table A3:** Statistical analysis of QTUG parameters with falls counts for PD1 dataset. M and F refer to population stratified by gender. U refers to Mann–Whitney rank sum statistic, ρ refers to Spearman’s correlation coefficient, while F refers to F-score from 1-way Anova test. Statistically significant differences (*p* < 0.05) are indicated by *.

	Mann–Whitney			Spearman			Anova		
Parameter Name	Faller (Mean ± std)	Non-Faller (Mean ± std)	U	ρ(All)	Ρ(M)	ρ(F)	F(All)	F (M)	F(F)
Mean step time (s)	0.62 ± 0.09	0.72 ± 0.10	42	−0.43	−0.19	−0.50	4.07 *	2.04	1.41
Mean stride length (cm/s)	107.00 ± 24.34	126.57 ± 22.17	48	−0.31	−0.05	−0.30	3.51	1.66	1.66
Stride time variability (%)	33.37 ± 12.92	22.63 ± 11.32	59	0.61 *	0.26	0.60	3.10	0.60	200.48 *
Walk ratio	1.09 ± 0.19	1.42 ± 0.26	42	−0.62 *	−0.64 *	−0.20	2.99	1.49	7.32
CV stride velocity (%)	47.41 ± 9.20	46.47 ± 4.89	51	0.10	−0.27	0.80	2.36	1.62	1.25
CV *Z*-axis ang. vel. (%)	107.12 ± 8.53	106.16 ± 8.00	53	−0.02	0.07	0.70	2.15	1.17	0.96
Mean swing time (s)	0.49 ± 0.05	0.52 ± 0.10	46	−0.44	−0.37	0.00	2.14	1.87	43.86 *
Double support variability (%)	55.00 ± 23.11	34.25 ± 23.59	59	0.53 *	0.45	0.00	2.00	2.86	6.00
Turn magnitude (deg/s)	64.87 ± 79.45	93.92 ± 121.88	52	−0.20	0.19	−1.00	1.90	9.22	29.05 *
Mean stride time (s)	1.32 ± 0.15	1.47 ± 0.20	43	−0.21	−0.19	−0.30	1.90	1.37	2.18
Step time asymmetry (%)	−24.30 ± 6.29	14.16 ± 22.16	36 *	−0.47	−0.58	−0.60	1.71	1.14	74.89 *
CV *Y*-axis ang. vel. (%)	104.85 ± 7.00	105.60 ± 2.95	47	−0.19	−0.49	0.70	1.59	1.21	0.63
Swing time variability (%)	19.06 ± 9.50	31.03 ± 14.75	45	−0.54 *	−0.48	−0.90	1.46	0.74	0.57
Min *X*-axis ang. vel. × Height (deg·m/s)	−656.15 ± 121.89	−730.02 ± 178.35	54	0.06	−0.09	−0.10	1.44	0.69	0.28
Time to Sit (s)	1.90 ± 0.62	2.15 ± 0.89	48	−0.37	−0.41	0.70	1.39	1.18	0.81
Single support variability (%)	18.02 ± 6.83	19.3 ± 27.26	51	0.09	0.06	0.10	1.36	3.13	4.60
CV stride length (%)	36.08 ± 8.45	33.73 ± 15.52	52	0.12	−0.09	0.50	1.22	0.68	1.75
Stance time variability (%)	42.50 ± 20.31	40.15 ± 6.90	56	0.46	0.30	0.50	1.12	2.72	0.53
Mean stance time (s)	0.82 ± 0.15	0.95 ± 0.18	45	−0.09	−0.01	−0.20	1.03	0.92	0.32
Stride time asymmetry (%)	−1.49 ± 12.09	−2.15 ± 5.26	50	−0.09	−0.02	0.10	1.03	3.91	0.44
Min *X*-axis ang. vel. (deg/s)	−378.73 ± 61.07	−422.86 ± 79.94	57	0.05	−0.03	0.30	1.02	0.67	0.74
Step time variability (%)	29.14 ± 13.03	25.62 ± 18.92	56	0.32	0.16	0.70	1.01	0.76	0.72
Mean stride velocity (cm/s)	80.55 ± 9.46	93.03 ± 13.17	46	−0.36	−0.28	−0.30	0.97	0.39	2.77
Mean double support	0.22 ± 0.05	0.29 ± 0.09	46	−0.04	−0.14	−0.20	0.95	0.62	5.27
Mean *Z*-axis ang. vel. (deg/s)	42.11 ± 8.15	43.37 ± 2.32	53	−0.04	−0.02	−0.10	0.95	1.28	0.73
Number of steps	13.63 ± 3.12	12.25 ± 3.63	55	0.20	0.03	−0.15	0.90	0.11	5.33
Number of gait cycles	6.63 ± 1.49	6.00 ± 1.87	55.5	0.20	0.07	−0.26	0.83	0.13	15.20
Cadence (steps/min)	91.50 ± 8.82	85.42 ± 8.77	58	0.13	−0.11	0.10	0.80	0.68	0.40
Magnitude mean at mid-swing points (deg/s)	220.23 ± 23.72	278.60 ± 47.40	40 *	−0.42	−0.39	−0.70	0.71	0.28	5.26
Mean *Z*-axis ang. vel. × Height (deg·m/s)	72.50 ± 13.64	74.14 ± 3.73	49	−0.13	0.06	0.30	0.68	1.01	0.97
Mean *Y*-axis ang. vel. × Height (deg·m/s)	100.46 ± 11.40	118.65 ± 8.36	40 *	−0.49	−0.31	−0.70	0.66	0.05	5.96
Swing time asymmetry (%)	−8.30 ± 9.65	9.29 ± 26.40	45	−0.18	−0.09	−0.70	0.60	0.52	15.59
Stride length asymmetry (%)	13.36 ± 21.82	4.65 ± 10.97	55	0.37	0.37	0.30	0.59	0.45	0.91
Min *Z*-axis ang. vel. (deg/s)	−212.79 ± 41.74	−211.98 ± 47.11	53	0.04	−0.15	0.10	0.58	0.53	4.90
Number of strides in turn	2.00 ± 0.50	2.25 ± 1.09	51	0.00	−0.24	0.71	0.57	0.44	0.20
Mean *Y*-axis ang. vel. (deg/s)	58.07 ± 5.23	69.93 ± 10.07	41	−0.50	−0.37	−0.90	0.54	0.08	17.40
Max *X*-axis ang. vel. (deg/s)	397.95 ± 136.75	329.90 ± 139.25	57	0.33	0.18	0.70	0.52	0.30	0.38
Max *X*-axis ang. vel. × Height (deg·m/s)	690.15 ± 241.87	574.03 ± 278.02	56	0.31	0.20	0.70	0.51	0.31	0.39
Max *Y*-axis ang. vel. × Height (deg·m/s)	522.79 ± 75.12	621.04 ± 143.92	47	−0.13	−0.03	−0.30	0.51	0.35	3.75
Walk time (s)	8.94 ± 1.84	8.53 ± 2.10	54	0.18	0.10	−0.30	0.50	0.02	5.61
Turn mid-point time (s)	5.99 ± 1.43	4.84 ± 1.40	59.	0.38	0.36	0.20	0.49	0.03	5.27
Magnitude range at mid-swing points (deg/s)	199.31 ± 40.43	198.76 ± 119.76	54	0.30	0.28	0.00	0.48	0.07	5.69
Return from turn time (s)	6.34 ± 0.87	6.55 ± 0.88	48	−0.06	−0.16	0.00	0.47	0.33	7.80
Ratio strides/turning time	0.67 ± 0.12	0.78 ± 0.43	54	−0.08	−0.19	0.30	0.46	0.24	0.10
Min *Z*-axis ang. vel. × Height (deg·m/s)	−367.52 ± 74.68	−363.26 ± 87.86	51	0.01	−0.15	0.10	0.45	0.42	6.45
Max *Z*-axis ang. vel. × Height (deg·m/s)	385.15 ± 86.89	434.88 ± 34.78	45	−0.21	−0.18	0.40	0.42	1.49	1.16
Min *Y*-axis ang. vel. × Height (deg·m/s)	−343.45 ± 72.88	−437.32 ± 119.89	58	0.19	−0.10	0.50	0.41	0.31	3.56
Turning time (s)	2.99 ± 0.54	3.00 ± 0.54	52	0.17	−0.09	0.50	0.41	0.23	0.90
Min *Y*-axis ang. vel. (deg/s)	−198.00 ± 38.24	−260.72 ± 90.53	58	0.11	−0.07	0.50	0.35	0.33	6.17
Time to stand (s)	2.07 ± 0.69	1.17 ± 0.31	63.5	0.52 *	0.48	0.72	0.35	0.18	0.74
Max *Z*-axis ang. vel. (deg/s)	223.24 ± 50.55	254.43 ± 21.03	45	−0.21	−0.18	0.00	0.33	1.55	1.87
TUG test time (s)	12.33 ± 2.11	11.39 ± 2.06	57	0.31	0.24	0.20	0.31	0.12	8.49
Max *Y*-axis ang. vel. (deg/s)	302.00 ± 37.09	368.15 ± 109.42	46	−0.08	−0.14	−0.30	0.25	0.23	6.58
Mean single support	0.39 ± 0.02	0.37 ± 0.06	57	−0.02	0.03	0.10	0.25	0.13	8.85
Stride velocity asymmetry (%)	10.85 ± 17.77	8.50 ± 18.30	54	0.08	−0.10	0.30	0.13	0.42	1.07
CV *X*-axis ang. vel. (%)	115.90 ± 8.89	117.16 ± 24.26	59	0.17	0.20	0.80	0.12	0.07	2.78
Stance time asymmetry (%)	−0.06 ± 18.66	−6.24 ± 22.11	57	0.13	0.35	0.80	0.09	0.93	2.74
Mean *X*-axis ang. vel. (deg/s)	54.77 ± 9.19	53.40 ± 6.21	52	0.10	0.24	0.00	0.09	0.13	0.09
Mean *X*-axis ang. vel. × Height (deg·m/s)	94.50 ± 15.62	90.80 ± 4.62	53	0.12	0.27	0.00	0.09	0.12	0.02

### Appendix B.3. PD2 Dataset

Analysis of the five mobility risk scores with falls counts for the PD2 dataset found non-significant associations between each mobility risk score and the number of falls as follows:

- Speed score, F = 2.09, *p* = 0.15
- Turn score, F = 0.71, *p*-value: 0.50
- Transfer score, F = 2.14, *p* = 0.14
- Variability score, F = 1.3, *p* = 0.29
- Symmetry score, F = 2.41, *p* = 0.11

Figure A1 below illustrates the association of each mobility risk score with falls counts for the PD2 dataset. Note: falls count data were only available for the count values 0, 1 and 3.

Table A4 below details results for a battery of statistical tests conducted on QTUG parameters calculated from the PD2 dataset (exercise intervention study of a sample of Parkinson’s Disease patients) [44]. Analyses examined statistical differences in each measure between fallers and non-fallers, as well as the relationship between each measure and the number of falls reported by participants. Significant associations were found between falls counts and a number of gait and mobility parameters.

**Table A4:** Statistical analysis of QTUG parameters with falls counts for PD2 dataset. M and F refer to population stratified by gender. U refers to Mann–Whitney rank sum statistic, ρ refers to Spearman’s correlation coefficient, while F refers to F-score from 1-way Anova test. Statistically significant differences (*p* < 0.05) are indicated by *.

	Mann–Whitney			Spearman			Anova		
Parameter Name	Faller (Mean ± std)	Non-Faller (Mean ± std)	U	ρ (All)	Ρ (M)	ρ (F)	F (All)	F (M)	F (F)
Min *X*-axis ang. vel. (deg/s)	−547.81 ± 150.96	−565.14 ± 93.27	119	0.07	0.44	−0.33	2.89	6.09 *	1.43
Ratio strides/turning time	1.36 ± 0.24	1.10 ± 0.29	142.5	0.41 *	0.39	0.40	2.81	2.87	1.08
Number of gait cycles	6.88 ± 1.45	5.39 ± 1.38	144.5 *	0.42 *	0.43	0.47	2.75	4.96 *	0.87
Min *X*-axis ang. vel. × Height (deg·m/s)	−927.44 ± 242.36	−972.68 ± 174.56	122	0.11	0.38	−0.33	2.66	4.28	1.60
Mean stride length (cm/s)	112.10 ± 12.10	126.20 ± 14.58	71 *	−0.41 *	−0.22	−0.60	2.55	1.32	1.63
Number of steps	14.13 ± 3.30	11.28 ± 2.66	143.5 *	0.41 *	0.41	0.52	2.46	5.18 *	1.05
Time to stand (s)	1.37 ± 0.37	1.00 ± 0.41	148 *	0.45 *	0.44	0.53	2.14	3.57	0.91
Stance time variability (%)	31.96 ± 16.77	41.87 ± 17.52	85	−0.28	−0.03	−0.40	1.73	0.02	0.59
Time to Sit (s)	2.10 ± 1.87	1.40 ± 0.46	118.5	0.08	0.60 *	−0.32	1.67	10.53 *	0.82
Turn mid-point time (s)	4.38 ± 1.35	3.47 ± 0.98	139	0.35	0.47	0.43	1.67	6.59 *	1.21
TUG test time (s)	10.04 ± 3.90	7.95 ± 1.93	137	0.32	0.54 *	0.43	1.63	9.33 *	0.35
Cadence (steps/min)	124.28 ± 14.23	114.54 ± 13.54	138	0.35	0.09	0.53	1.57	0.00	0.32
Step time asymmetry (%)	−5.28 ± 8.07	4.56 ± 15.91	85	−0.23	−0.44	0.16	1.52	2.77	0.17
Double support variability (%)	44.87 ± 22.83	59.47 ± 24.87	80		−0.06		1.44	0.00	1.21
Return from turn time (s)	5.66 ± 2.73	4.48 ± 1.05	124	0.16	0.54 *	0.00	1.43	8.86 *	0.04
Single support variability (%)	14.63 ± 6.29	15.72 ± 8.02	105	−0.07	0.09	−0.33	1.40	0.27	1.18
Number of strides in turn	2.75 ± 0.66	2.28 ± 0.65	131.5	0.30	0.51 *	0.30	1.36	5.41 *	1.00
Min *Z*-axis ang. vel. × Height (deg·m/s)	−344.60 ± 89.50	−428.51 ± 142.38	133	0.26	0.54 *	−0.05	1.35	4.26	0.09
Mean stride time (s)	0.99 ± 0.13	1.09 ± 0.15	75	−0.38	−0.16	−0.48	1.29	0.05	0.23
Min *Z*-axis ang. vel. (deg/s)	−204.88 ± 60.36	−248.61 ± 78.34	133	0.25	0.63 *	−0.05	1.20	5.03 *	0.04
Mean *Z*-axis ang. vel. × Height (deg·m/s)	58.29 ± 21.38	73.53 ± 22.58	79	−0.32	−0.60 *	−0.09	1.16	5.47 *	0.23
CV *Y*-axis ang. vel. (%)	102.25 ± 12.24	99.47 ± 4.61	111	0.00	0.66 *	−0.48	1.09	26.42 *	0.33
Max *X*-axis ang. vel. (deg/s)	513.96 ± 130.46	521.80 ± 115.13	108	0.04	−0.13	0.26	1.08	0.24	0.99
Mean stance time (s)	0.54 ± 0.12	0.63 ± 0.14	84	−0.27	−0.06	−0.17	1.05	0.12	0.06
Max *Y*-axis ang. vel. × Height (deg·m/s)	695.89 ± 97.81	754.29 ± 87.17	87	−0.24	−0.28	−0.21	1.02	3.30	0.31
Mean *Z*-axis ang. vel. (deg/s)	34.69 ± 13.72	42.57 ± 12.00	81	−0.30	−0.66 *	−0.02	0.97	6.72 *	0.28
Mean *Y*-axis ang. vel. × Height (deg·m/s)	151.04 ± 34.21	164.50 ± 22.61	97	−0.10	−0.54 *	0.00	0.95	7.95 *	0.00
Walk time (s)	6.97 ± 2.05	5.96 ± 1.43	128	0.23	0.41	0.36	0.94	5.11 *	0.36
Mean step time (s)	0.47 ± 0.05	0.50 ± 0.05	83	−0.28	−0.03	−0.29	0.94	0.01	0.45
Mean *X*-axis ang. vel. × Height (deg·m/s)	123.93 ± 36.45	134.50 ± 26.58	95	−0.11	−0.57 *	0.24	0.91	6.50 *	0.08
Stride time variability (%)	18.36 ± 10.00	24.30 ± 12.92	93	−0.19	−0.03	−0.09	0.90	0.04	0.12
Max *X*-axis ang. vel. × Height (deg·m/s)	871.44 ± 218.31	900.28 ± 220.22	100	−0.05	−0.09	0.26	0.88	0.12	1.13
Mean single support	0.47 ± 0.06	0.44 ± 0.05	125	0.17	0.06	−0.14	0.82	0.11	0.35
Mean *X*-axis ang. vel. (deg/s)	73.60 ± 23.74	78.21 ± 14.57	99	−0.07	−0.60 *	0.24	0.77	9.83 *	0.03
Walk ratio	1.26 ± 0.28	1.32 ± 0.20	87	−0.26	0.09	−0.77 *	0.76	0.63	2.77
Mean stride velocity (cm/s)	110.73 ± 11.71	118.38 ± 15.12	86	−0.24	−0.31	−0.60	0.75	1.80	1.66
Max *Y*-axis ang. vel. (deg/s)	410.66 ± 64.62	439.30 ± 46.89	100	−0.10	−0.41	−0.28	0.73	6.76 *	0.61
Step time variability (%)	14.55 ± 3.67	18.03 ± 7.57	93	−0.16	−0.16	0.05	0.69	0.72	0.04
Mean *Y*-axis ang. vel. (deg/s)	89.57 ± 22.92	95.83 ± 12.59	102	−0.05	−0.57 *	−0.16	0.67	12.42 *	0.05
Magnitude range at mid-swing points (deg/s)	231.85 ± 38.68	262.60 ± 68.58	92	−0.17	−0.16	−0.38	0.63	0.37	0.89
Max *Z*-axis ang. vel. x Height (deg·m/s)	370.38 ± 138.85	439.48 ± 145.56	85	−0.25	−0.54 *	−0.09	0.57	4.23	0.48
Mean swing time (s)	0.45 ± 0.05	0.46 ± 0.05	92	−0.20	0.06	−0.47	0.51	0.04	0.97
Swing time asymmetry (%)	−1.15 ± 6.95	5.01 ± 15.92	93	−0.17	−0.25	−0.12	0.50	0.76	0.04
Mean double support	0.11 ± 0.06	0.15 ± 0.08	94	−0.16	−0.03	−0.10	0.45	0.02	0.11
Max *Z*-axis ang. vel. (deg/s)	220.89 ± 90.20	254.29 ± 77.16	88	−0.22	−0.57 *	0.05	0.42	5.17 *	0.54
Min *Y*-axis ang. vel. × Height (deg·m/s)	−448.05 ± 67.60	−505.38 ± 184.20	119	0.11	0.06	0.26	0.34	0.26	0.62
Min *Y*-axis ang. vel. (deg/s)	−263.50 ± 40.48	−293.17 ± 98.47	119	0.11	0.06	0.46	0.32	0.41	0.90
CV *X*-axis ang. vel. (%)	112.51 ± 11.99	114.59 ± 8.75	107	−0.04	0.25	−0.12	0.25	0.58	0.01
CV *Z*-axis ang. vel. (%)	114.45 ± 10.74	116.44 ± 10.80	97	−0.10	−0.31	−0.03	0.25	0.43	0.13
Swing time variability (%)	17.52 ± 7.16	19.20 ± 9.93	101	−0.09	0.09	−0.38	0.25	0.02	0.46
Magnitude mean at mid-swing points (deg/s)	319.85 ± 61.43	331.20 ± 33.43	111	0.03	−0.35	−0.09	0.22	5.54 *	0.31
Stride length asymmetry (%)	−8.04 ± 14.32	−8.47 ± 19.78	112	0.03	−0.22	0.13	0.21	0.75	1.49
Turning time (s)	2.05 ± 0.41	2.10 ± 0.48	108	−0.02	0.28	0.07	0.16	0.67	0.06
CV stride velocity (%)	39.37 ± 8.67	38.05 ± 12.71	119	0.11	0.31	−0.15	0.11	0.63	0.06
Stride velocity asymmetry (%)	−5.78 ± 11.33	−3.46 ± 17.00	102	−0.09	−0.09	−0.16	0.10	0.47	0.17
Turn magnitude (deg/s)	141.09 ± 143.28	135.91 ± 104.43	101	−0.09	−0.44	0.05	0.09	2.61	0.37
Stance time asymmetry (%)	−5.88 ± 22.93	−10.84 ± 29.75	116	0.09	0.09	0.05	0.09	0.25	0.09
CV stride length (%)	31.76 ± 10.14	34.03 ± 13.32	106	−0.01	0.25	−0.13	0.08	0.41	1.05
Stride time asymmetry (%)	−4.09 ± 12.40	−5.59 ± 16.93	111	0.03	0.06	−0.07	0.03	0.06	0.08

## Appendix C. Model Training Performance

Training set results for Poisson regression models for the TD dataset are detailed in Table A5 below. The results are reported with outliers removed (number of falls >10), resulting in three samples being excluded from the training sets.

**Table A5:** Training set results for each Poisson regression model, selected using an elastic net procedure. Outliers (*NumFalls* > 10) are removed to improve model fit.

**All**	**N**	**R^2^**	**RMSE**	**ρ**	**#Features**
TD-All	1015	0.08	0.46	0.29	16
TD-Fallers	347	0.03	0.51	0.19	17
TD-PD	29	0.25	0.57	0.62	0
TD-Fallers-PD	19	0.04	0.69	0.33	0
TD-NoPD	986	0.08	0.45	0.30	17
TD-Fallers-NoPD	328	0.04	0.49	0.21	19
Ensemble-TD -PD	29	0.07	0.63	0.24	2
**Male**	**N**	**R^2^**	**RMSE**	**ρ**	**#Features**
TD -All	344	0.00	0.46	0.29	3
TD-Fallers	86	0.02	0.58	0.27	5
TD-PD	18	0.04	0.49	0.64	3
TD-Fallers-PD	11	0.10	0.42	0.83	10
TD-NoPD	326	0.11	0.42	0.38	19
TD-Fallers-NoPD	75	0.09	0.57	0.33	12
Ensemble-TD-PD	18	0.04	0.49	0.09	2
**Female**	**N**	**R^2^**	**RMSE**	**ρ**	**#Features**
TD-All	671	0.07	0.47	0.27	11
TD-Fallers	261	0.08	0.47	0.28	17
TD-PD	11	0.03	0.78	0.76	3
TD-Fallers-PD	8	0.63	0.52	0.85	19
TD-NoPD	660	0.05	0.46	0.26	9
TD-Fallers-NoPD	253	0.08	0.44	0.27	16
Ensemble-TD-PD	18	0.23	0.70	0.50	2

Figure A2 below shows the elastic net trace and deviance plots for TD-All model fit. The selected model chosen was for the lambda value yielding the minimum standard error (SE) for a minimum model size of 3 and a maximum model size of 20.

Figure A3 below shows the predicted falls count values against the actual number of falls for the selected Poisson elastic net model trained using TD-All dataset.

## Appendix D. Model Coefficients

Regression coefficients and the selected feature for each model trained on TD data are detailed in the tables below (Table A6, Table A7, Table A8, Table A9, Table A10, Table A11 and Table A12). Each set of model coefficients is identified by the TD data subset used to train it.

**Table A6:** TD-All falls count model coefficients. Beta refers to Poisson regression coefficients, chosen through an elastic net procedure.

All		Male		Female	
Beta	Features	Beta	Features	Beta	Features
0.268767	Intercept	−1.43612	Intercept	−0.48572	Intercept
−0.71395	single_support	0.004605	TurnTime	−0.02719	single_support
−0.00214	swing_CV	−0.00196	AV_AP_CV	−0.00117	swing_CV
−0.01688	AV_ML_CV	−0.00319	AV_V_CV	0.035354	TurnTime
0.049997	TurnTime			0.001368	TurnEndTime
−0.02888	AV_AP_CV			−0.00102	AV_AP_CV
−0.03023	AV_V_CV			−0.00245	AV_V_CV
9.38 × 10^−5^	AV_V_min			0.000345	AV_V_min
−0.00293	MeanVelocity			−0.00183	MeanVelocity
−0.00191	VelocityCV			−0.00354	MeanStrideLen
−0.00544	MeanStrideLen			0.000317	AV_V_minByH
0.000265	AV_V_minByH			0.005656	ManualTUG
0.003158	ManualTUG				

**Table A7:** TD-Fallers falls count model coefficients. Beta refers to Poisson regression coefficients, chosen through an elastic net procedure.

All		Male		Female	
Beta	Features	Beta	Features	Beta	Features
−0.61432	Intercept	−0.81756	Intercept	0.678357	Intercept
−3.9 × 10^−5^	AV_ML_max	0.000941	GaitCycles	−0.00501	single_support
0.000163	AV_ML_min	−7.5 × 10^−6^	AV_mid_swing_mean	−0.07407	single_support_CV
0.000448	GaitCycles	−0.00058	AV_turn_mag	−0.0037	swing_CV
−7 × 10^−5^	AV_mid_swing_mean	−8 × 10^−5^	MeanVelocity	−0.00023	Cadence
−0.00021	AV_turn_mag	0.00018	ManualTUG	−4.4 Page: 23 × 10^−5^	AV_turn_mag
−0.00013	AV_V_max			−0.00302	AV_V_mean
−0.00091	MeanVelocity			−0.00041	AV_V_max
−0.00084	MeanStrideLen			0.000281	AV_V_min
−0.00016	AV_ML_maxByH			−0.02218	VelocityCV
0.000205	AV_ML_minByH			−0.00524	MeanStrideLen
−6.5 × 10^−5^	AV_V_maxByH			−0.03254	StrideLenCV
0.007641	ManualTUG			−3.3 × 10^−5^	AV_ML_maxByH
				0.000551	AV_ML_minByH
				−0.0028	AV_V_meanByH
				−0.00051	AV_V_maxByH
				0.000429	AV_V_minByH
				0.007744	ManualTUG

**Table A8:** TD-PD falls count model coefficients. Beta refers to Poisson regression coefficients, chosen through an elastic net procedure.

All		Male		Female	
Beta	Features	Beta	Features	Beta	Features
−1.34037	Intercept	−0.9424	Intercept	−0.01892	Intercept
−0.15412	single_support_CV	0.037872	stance_CV	−3.2 × 10^−5^	AV_AP_max
0.009744	stance_CV	0.067785	swing_CV	0.010441	MeanTurningTime
0.113734	swing_CV	6.52 × 10^−5^	AV_ML_maxByH	−4 × 10^−5^	AV_AP_maxByH
−0.07459	step_CV				
0.001598	AV_ML_max				
0.093939	AV_ML_CV				
−0.00088	AV_turn_mag				
−0.02979	TurnTime				
0.081551	AV_AP_CV				
−0.00035	AV_AP_mean				
−0.00057	AV_AP_max				
0.189167	AV_V_CV				
−0.16128	StrideLenCV				
0.055812	MeanStepsTurn				
9.12 × 10^−5^	AV_ML_maxByH				
−0.0004	AV_AP_meanByH				
−0.00035	AV_AP_maxByH				
−8.6 × 10^−5^	AV_V_maxByH				

**Table A9:** TD-Fallers-PD falls count model coefficients. Beta refers to Poisson regression coefficients, chosen through an elastic net procedure.

All		Male		Female	
Beta	Features	Beta	Features	Beta	Features
−2.00125	Intercept	−1.3143	Intercept	0.241844	Intercept
0.316766	AV_V_CV	−0.00523	single_support_CV	0.92493	double_support
0.000114	AV_ML_minByH	0.380233	swing	−0.53451	single_support
0.000257	ManualTUG	0.095187	swing_CV	−1.63885	swing
		−0.00655	GaitCycles	−0.14224	step_CV
		−0.00101	StepNo	0.000438	AV_ML_min
		0.001431	AV_V_CV	0.031803	GaitCycles
		4.5 × 10^−5^	AV_V_max	0.00999	StepNo
		−0.00066	MeanStepsTurn	−0.00067	AV_AP_mean
		4.82 × 10^−5^	AV_V_maxByH	0.070045	AV_V_CV
		−0.00479	ManualTUG	−0.00041	AV_V_mean
				−0.00155	MeanStrideLen
				0.1255	MeanTurningTime
				0.078945	MeanStepsTurn
				0.115859	MeanTurnRatio
				−0.00015	AV_ML_meanByH
				0.000433	AV_ML_minByH
				−0.00052	AV_AP_meanByH
				−1.4 × 10^−5^	AV_AP_maxByH
				−0.00048	AV_V_meanByH

**Table A10:** TD-NoPD falls count model coefficients. Beta refers to Poisson regression coefficients, chosen through an elastic net procedure.

All		Male		Female	
Beta	Features	Beta	Features	Beta	Features
0.065301385	Intercept	−0.27568	Intercept	−0.7974	Intercept
0.018407619	double_support	−0.64365	single_support	0.000294	WalkTime
−0.636511776	single_support	0.012447	single_support_CV	0.001582	GaitCycles
−0.019776173	AV_ML_CV	0.106376	stance	0.027008	TurnTime
−0.000217534	Cadence	0.455945	stride	0.001196	TurnEndTime
0.051661245	TurnTime	−0.07504	AV_ML_CV	0.000128	AV_V_min
−0.028394423	AV_AP_CV	−0.00496	Cadence	−0.00115	MeanVelocity
−0.030063354	AV_V_CV	−0.00126	AV_turn_mag	−0.00228	MeanStrideLen
1.30698 × 10^−5^	AV_V_min	0.05316	TurnTime	0.00015	AV_V_minByH
−0.00240226	MeanVelocity	−0.08651	AV_AP_CV	0.003563	ManualTUG
−0.000603359	VelocityCV	−0.0796	AV_V_CV		
−0.005019106	MeanStrideLen	0.005054	AV_V_mean		
0.000201194	AV_V_minByH	0.000411	AV_V_max		
0.003828431	ManualTUG	−0.0005	AV_V_min		
		−0.00456	MeanVelocity		
		−0.00429	MeanStrideLen		
		0.001651	AV_V_meanByH		
		4.84 × 10^−5^	AV_V_maxByH		
		−0.00022	AV_V_minByH		
		0.011925	ManualTUG		

**Table A11:** TD-Fallers-NoPD falls count model coefficients. Beta refers to Poisson regression coefficients, chosen through an elastic net procedure.

All		Male		Female	
Beta	Features	Beta	Features	Beta	Features
−0.360429	Intercept	−0.58931	Intercept	0.636402	Intercept
−0.001329	stance_CV	−0.52148	single_support	−0.05248	single_support_CV
−5.32 × 10^−5^	AV_ML_max	−0.1655	swing	−0.00303	stride_CV
2.381 × 10^−5^	AV_ML_min	0.002619	WalkTime	−0.00172	Cadence
−0.003735	AV_ML_CV	0.030711	GaitCycles	0.000913	TurnEndTime
0.001525	WalkTime	0.01269	StepNo	−0.00126	AV_V_mean
0.0003581	GaitCycles	−0.00046	AV_mid_swing_mean	−0.0004	AV_V_max
−0.000813	Cadence	−0.00163	AV_turn_mag	0.000391	AV_V_min
−0.000133	AV_mid_swing_mean	−6.4 × 10^−5^	AV_AP_mean	−0.03916	VelocityCV
−0.00028	AV_turn_mag	−0.00157	MeanVelocity	−0.00515	MeanStrideLen
−0.002004	AV_AP_CV	−0.01273	MeanTurnRatio	−0.03979	StrideLenCV
−0.003814	AV_V_CV	−0.00011	AV_AP_meanByH	−0.00012	AV_ML_maxByH
−0.000311	AV_V_max	0.003843	ManualTUG	0.000233	AV_ML_minByH
−0.001205	MeanVelocity			−0.00161	AV_V_meanByH
−0.006212	VelocityCV			−0.00045	AV_V_maxByH
−0.001575	MeanStrideLen			0.000453	AV_V_minByH
−0.000173	AV_ML_maxByH			0.006292	ManualTUG
0.0001248	AV_ML_minByH				
−0.000177	AV_V_maxByH				
0.0083301	ManualTUG				

**Table A12:** Ensemble model coefficient for combining TD-NoPD and TD-Fallers-NoPD models.

All		Male		Female	
Beta	Dataset	Beta	Dataset	Beta	Dataset
−6.3744604	TD-NoPD	−2.08471	TD-NoPD	−7.93649	TD-NoPD
4.90488203	TD-Fallers-NoPD	−0.27718	TD-Fallers-NoPD	8.236352	TD-Fallers-NoPD
